# Progression of HIV Disease Among Patients on ART in Ethiopia: Application of Longitudinal Count Models

**DOI:** 10.3389/fpubh.2019.00415

**Published:** 2020-02-19

**Authors:** Belay Desyebelew Andualem, Birhanu Teshome Ayele

**Affiliations:** ^1^Department of Statistics, Dire Dawa University, Dire Dawa, Ethiopia; ^2^Department of Statistics, Addis Ababa University, Addis Ababa, Ethiopia; ^3^Division of Epidemiology and Biostatistics Unit, Faculty of Medicine and Health Sciences, Stellenbosch University, Stellenbosch, South Africa

**Keywords:** HIV/AIDS, CD4 count, longitudinal data, Poisson-Normal model, Poisson-Gamma-Normal model, antiretroviral therapy (ART), Ethiopia, Debre Markos

## Abstract

Although the world has been fighting HIV disease in unity and patients are getting antiretroviral therapy treatment, HIV disease continues to be a serious health issue for some parts of the world. A large number of AIDS-related deaths and co-morbidities are registered every year in resource-limited countries like Ethiopia. Most studies that have assessed the progression of the disease have used models that required a continuous response. The main objective of this study was to make use of appropriate statistical models to analyze routinely collected HIV data and identify risk factors associated with the progression of the CD4^+^ cell count of patients under ART treatment in Debre Markos Referral Hospital, Ethiopia. In this longitudinal retrospective study, routine data of 445 HIV patients registered for ART treatment in the Hospital were used. As overdispersion was detected in the data, and Poisson-Gamma, Poisson-Normal, and Poisson-Gamma-Normal models were applied to account for overdispersion and correlation in the data. The Poisson-Gamma-Normal model with a random intercept was selected as the best model to fit the data. The findings of the study revealed the time on treatment, sex of patients, baseline WHO stage, and baseline CD4^+^ cell count as significant factors for the progression of the CD4^+^ cell count.

## 1. Introduction

HIV disease continues to be a serious health issue for resource-limited countries like Ethiopia. According to the UNAIDS (2016) fact sheet, there were about 2.1 million new cases of HIV in 2015 globally (1). About 36.7 million people were living with HIV around the world, and, as of June 2016, 18.2 million people living with HIV were receiving medicine to treat HIV, called antiretroviral therapy (ART). An estimated 1.1 million people died from AIDS-related illnesses in 2015, and 35 million people have died from AIDS-related illnesses since the start of the epidemic. CD4^+^ cell counts are the primary targets of HIV. The relentless destruction of CD4^+^ cell counts by HIV, either directly or indirectly, results in the loss of HIV-specific immune responses and, finally, non-specific immune response in the AIDS stage. The estimation of peripheral CD4^+^ cell counts has been used as a tool for monitoring disease progression and the effectiveness of antiretroviral treatment (ART) (2). The changes in the CD4^+^ cell counts are important indicators of the response to ART. Initial CD4^+^ cell count, age, gender, smoking, unemployment, WHO stage, hospital, opportunistic infections, body mass index, changing doctors during outpatient follow up, use of alcohol and drugs, and duration of treatment (in months) are some of the significant determinants that affect CD4^+^ cell count progression of patients on ART (3–5).

Most studies conducted in the area fitted statistical models that require (multivariate) Normal distribution by considering CD4^+^ cell counts as continuous variable. When this assumption is violated, even after transformation, considering Poisson-related models is a natural choice. One of the common problems one can be faced with in analyzing count data like the CD4^+^ cell count is overdispersion. A Negative Binomial model can be considered to overcome this problem. Trindade et al. applied Poisson and Negative Binomial models using the multilevel (ML) approach and the generalized estimations equations (GEE) to model CD4^+^ cell counts of 587 HIV seropositive patients, and they stated that the best marginal model to fit the data was the Negative Binomial (NB) with an exchangeable correlation structure (6). Tekle et al. also employed different count data analysis methods starting from the ordinary Poisson regression model to study CD4^+^ cell counts of 222 HIV positive patients, and they found that Poisson-Normal-Gamma is the best model to fit their data (7). In this study, we applied various count data models to study the progression of the CD4^+^ cell count of HIV patients and identified risk factors for progression of patients' CD4^+^ cell count in Debre Markos Referral Hospital, Ethiopia.

## 2. Materials and Methods

In practice, it is common to have response variables of a count type-like number of the CD4^+^ cell count in a cubic milliliter of blood. Some data analysts treat the CD4^+^ cell count as a continuous measure and apply the linear mixed effects model. But that practice ignores two facts: the data are really discrete, and the distributions of count variables are usually skewed. For these reasons, the use of models that assume (multivariate) normality might not be efficient (8). Even if the data is transformed and these models are applied, the interpretation might not be straightforward. In scenarios like this, it is better to apply statistical models that account for the nature of the data.

Our data includes 445 HIV-positive patients who started ART treatment between December 2005 and July 2014 in Debre Markos Referral Hospital, Ethiopia. The minimum number of measurements was two and the maximum was seven. Patients with less than two measurements and age of <15 years were excluded from the study.

For our data, the assumption of multivariate normality failed, and this suggested that use of a linear mixed model was not appropriate (Table 1). The Poisson regression model with normal random effects and models that account for both correlation between repeated measures and overdispersion simultaneously were thus considered in line with Booth et al. (9) and Molenberghs et al. (10, 11).

**Table 1:** Multivariate normality test.

**Test**	**Value**	***P*-value**	**Result**
Henze-Zirkler's test	1.099	< 0.0001	Data are not multivariate normal
Mardia's test			Data are not multivariate normal
Skewness (22.51)	138.842	0.0002	
Kurtosis (71.24)	2.231	0.0257	
Royston's test	25.294	0.0002	Data are not multivariate normal

### 2.1. Variables in the Study

#### 2.1.1. Dependent Variable

The dependent variable of this study was the CD4^+^ cell count per cubic millimeter of blood of HIV-infected patients who are under ART treatment.

#### 2.1.2. Independent Variables

The independent variables considered in this study were selected based on related literature (5, 7). These include the sex of patients, age of patients (age at the initiation of the treatment), baseline CD4^+^ cell count (the CD4^+^ cell count of the patients at the start of the treatment), WHO clinical stage at baseline (stage I, stage II, stage III, and stage IV), marital status at baseline, baseline weight, level of education at baseline, functional status at baseline, TB status at baseline, and time in months. Functional status was defined as WHO categories: Ambulatory and Working. Patients who are able to perform activities of daily living but not able to work or play are classified as ambulatory and the who are able to perform usual work in or out of the house, harvest, go to school or for children, normal activities, or playing were classified as working.

### 2.2. Poisson Model

Let *Y*_*i*_ be the ith CD4^+^ cell count and is Poisson distributed with mean λ_*i*_. The density function of *Y*_*i*_ can then be written as

(1) $$\begin{array}{r} f\left(Y_{i}=y_{i}|\lambda_{i}\right)=\frac{e^{-\lambda_{i}}\lambda_{i}^{y_{i}}}{y_{i}!}=exp\left\{y_{i}\ln\lambda_{i}-\lambda_{i}-\ln y_{i}!\right\}, \end{array}$$

The Poisson distribution belongs to the exponential family, with natural parameter θ_*i*_ equal to lnλ_*i*_, scale parameter ϕ = 1, and variance function *v*(λ_*i*_) = λ_*i*_ (12). The logarithm is the natural link function, leading to the classical Poisson regression model *Y*_*i*_ ~ *Poisson*(λ_*i*_), with $\log\left(\lambda_{i}\right)=X_{i}^{T}\beta$.

### 2.3. Poisson-Gamma Model

The standard Poisson distribution requires the mean and variance to be equal. When this assumption fails, the Poisson-Gamma model should be used to fit the data. Assume that *Y*_*i*_|θ_*i*_ ~ *Poi*(θ_*i*_λ_*i*_), where θ_*i*_ denotes an independent and identically distributed (iid) sample of unit mean Gamma random variables with shape parameter α (9). Conditional on θ_*i*_, the CD4 count of the *i*th patient follows a Poisson distribution with mean θ_*i*_λ_*i*_. The counts are then marginally independent Poisson-Gamma random variables [*Y*_*i*_ ~NB(α, λ_*i*_)] with mean λ_*i*_ and variance $\lambda_{i}+\lambda_{i}^{2}/\alpha$. Hence, the parameter α quantifies the amount of overdispersion with α = ∞ corresponding to no overdispersion with respect to the Poisson distribution. The mass function of the Poisson-Gamma random variables is given by

(2) $$\begin{array}{r} Pr\left(Y_{i}=y;\alpha ,\lambda_{i}\right)=\frac{\Gamma \left(y+\alpha \right)}{\Gamma \left(\alpha \right)y!}{\left(\frac{\alpha }{\lambda_{i}+\alpha }\right)}^{\alpha }{\left(\frac{\lambda_{i}}{\lambda_{i}+\alpha }\right)}^{y} \end{array}$$

The Poisson-Gamma model (also known as the Negative Binomial model) is given by $\log\left(\lambda_{i}\right)=X_{i}^{T}\beta$.

### 2.4. Poisson-Normal Model

For μ_*ij*_ =E(*Y*_*ij*_|*b*_*i*_) and known link function η(.), the generalized linear mixed model can be expressed as:

(3) $$\begin{array}{r} \eta \left(\mu_{ij}\right)=\eta \left[E\left(Y_{ij}|b_{i}\right)\right]=X_{ij}^{T}\beta +Z_{ij}^{T}b_{i} \end{array}$$

where *Y*_*ij*_ is the CD4^+^ cell count of the *i*th patient at *j*th visit (measurement). β= a p-dimensional vector of unknown fixed regression coefficients. *b*_*i*_ = a q-dimensional vector of unknown random regression coefficients for the ith individual, and these are often assumed to be drawn independently from the N(0, D), and D is the variance-covariance matrix of the random effects. *X*_*ij*_ and *Z*_*ij*_ are p-dimensional and q-dimensional vectors of known covariate values, respectively (10). The generalized mixed Poisson model with normal random effects (Poisson-Normal model) becomes

(4) $$\begin{array}{r} \ln\left(\lambda_{ij}\right)=X_{ij}^{T}\beta +Z_{ij}^{T}bi \end{array}$$

This model is referred to as the Poisson-Normal model because it assumes Poisson distribution for the counts and normal distribution for the random effects *b*_*i*_ (10, 11).

### 2.5. Poisson-Gamma-Normal Model

According to Molenberghs et al. (10, 11), a model combining the ideas from the Poisson-Normal and overdispersion models for repeated Poisson data with overdispersion can be specified as follows *Y*_*ij*_ ~ *poi*(θ_*ij*_λ_*ij*_)

(5) $$\begin{array}{r} \lambda_{ij}=\exp\left(X_{ij}^{T}\beta +Z_{ij}^{T}b_{i}\right) \end{array}$$

where θ_*ij*_ capture overdispersion and denote an independent and identically distributed (iid) sample of unit mean gamma random variables with shape parameter α and scale parameter β=1/α, and where *b*_*i*_ ~ *N*(0, *D*) and θ_*ij*_ ~ *Gamma*(α, β). This model is called the Poisson-Gamma-Normal (combined) model because it includes both Normal (*b*_*i*_) and Gamma (θ_*ij*_) random effects to account for correlation and overdispersion, respectively.

### 2.6. Methods of Parameter Estimation

In this study, we used glmer and glmer.nb functions in R under packages MASS and lme4. A Laplace approximation was used to obtain parameter estimates. The R code used to fit the models is available in Supplementary Material.

### 2.7. Model Comparison

To select the important variables, first the main effect, main effect by time interaction, and plausible main effect by main effect interactions were incorporated to the initial candidate models, the non-significant interaction effects were then removed, and the models were refitted again and so on. The best model that can fit the data was selected using various information criteria (AIC, BIC, and −2loglikelihood) (**Table 7**). The model with smallest values of information criteria was selected as the final model.

## 3. Results and Discussion

### 3.1. Descriptive Analysis

In this section, CD4^+^ cell count data obtained from 445 HIV patients on ART treatment in Debre Markos Referral Hospital were summarized. The majority of the HIV patients [347 (78.0%)] started antiretroviral treatment with CD4^+^ cell counts <200 cells/mm^3^. At the start of the treatment, the median CD4^+^ cell count of the patients was 145 CD4^+^ cells/mm^3^ of blood with IQR of 107.00 CD4^+^ cells/*mm*^3^ of blood. The minimum and maximum baseline CD4^+^ cell counts were three and 971 CD4^+^ cell cells/mm^3^ of blood, respectively.

The summary of CD4^+^ cell counts at different time points is given in Table 2. As can be seen in Table 2, the median CD4^+^ cell count increased over time. The IQR of CD4^+^ cell counts increased at some points and then started to decrease after the 24th month. The number of patients decreased at some points and increased at others, which implies the presence of intermittent missingness in the data. That means some patients were falling out of care and then re-engaging, or they did not have CD4^+^ cell counts that were spaced perfectly every 6 months.

**Table 2:** Summary of CD4^+^ cell count at different time points.

Time	0	6	12	18	24	30	36
*n*	445	372	320	283	271	279	261
Median	145	260	302	323	342	357	371
IQR (Q1, Q3)	107 (85, 192)	206.25 (177, 383.25)	201.5 (204.5, 406)	258.5 (216.5, 475)	274 (234.5, 508.5)	220.5 (261, 481.5)	209 (272, 481)

Data on demographic and clinical characteristics of the patients was collected at the start of antiretroviral treatment. Among the 445 patients, 280 (62.9%) were females. The male patients had a 134.84 mean baseline CD4^+^ cell count, while the female patients had a mean baseline CD4^+^ cell count of 168.91. On average, female patients started ART treatment at a relatively higher CD4^+^ cell count. The difference in mean CD4^+^ cell count of the two groups increases as time increases. The average CD4^+^ cell count of females was higher than males at all time points and the difference increases over time.

WHO stage III had a higher number of patients [282 (63.4%)] as compared to the other three stages. WHO stage II took second place in number of patients [77 (17.3%)], and WHO stage IV had the smallest number of patients [27 (6.1%)]. As expected, patients on WHO stage I had a higher CD4^+^ cell count at all time points as compared to patients of the other three stages of the disease.

Patients with a working functional status have a higher mean CD4^+^ cell count at all time points than that of patients with ambulatory functional status. Among the 445 HIV patients included in this study, 346 (77.8%) were patients with working functional status and 99 (22.3%) were ambulatory (Table 3). About 27.3% of the 445 HIV patients were TB positive at baseline. TB negative patients had a higher mean CD4^+^ cell count at all time points compared to TB positive patients, implying the impact of the HIV-TB coinfection.

**Table 3:** Summary of CD4^+^ cell count progression for some categorical covariates.

		**Time (in months)**							
**Covariates**	**Categories**		**0**	**6**	**12**	**18**	**24**	**30**	**36**
Sex									
	Male	*n*	165	131	117	100	97	106	106
		Median	130.0	235.0	250.0	295.5	297.0	312.0	318.0
		IQR (Q1, Q3)	112 (62, 174)	177 (165, 342)	187 (172, 359)	255.25 (170, 425.25)	270 (181, 451)	209.5 (210.25, 419.75)	188.75 (238, 426.75)
	Female	*n*	280	241	203	183	174	173	155
		Median	155.5	275.0	324.0	331.0	376.5	377.0	418.0
		IQR (Q1, Q3)	110 (92, 202)	204 (191, 395)	216 (227.5, 443.5)	258.5 (236, 494.5)	293.5 (257, 550.5)	226 (289, 515)	250.5 (301.5, 552)
WHO stage									
	Stage I	*n*	59	51	41	36	38	35	36
		Median	187.0	300.0	349.0	444.5	446.0	317.0	396.0
		IQR (Q1, Q3)	145.5 (131, 276.5)	283 (216.5, 499.5)	308 (243, 551)	288.25 (326.5, 614.75)	333.75 (257, 590.75)	209 (285, 494)	290.5 (323.5, 614)
	Stage II	*n*	77	65	54	52	47	48	42
		Median	130.0	229.0	267.5	261.5	297.0	351.5	332.0
		IQR (Q1, Q3)	96 (83, 179)	187 (156, 343)	160.75 (189.5, 350.25)	214.25 (158.75, 373)	229.5 (175, 404.5)	193.25 (251.5, 444.75)	164.75 (245, 409.75)
	Stage III	*n*	282	235	208	175	171	173	165
		Median	143.0	258.0	314.0	322.5	342.0	363.0	381.0
		IQR (Q1, Q3)	106.75 (83.25, 190)	180.75 (179.25, 360)	199 (206, 405)	268 (202.25, 470.25)	255.5 (243.5, 499)	225 (256, 481)	211.5 (262, 473.5)
	Stage IV	*n*	27	21	17	20	15	23	18
		Median	114.0	319.5	246.5	343.0	380.0	361.0	434.0
		IQR (Q1, Q3)	123 (49.5, 172.5)	276 (150.75, 426.75)	200.75 (153.75, 354.50)	263 (230, 493)	376.5 (219, 595.5)	229 (257, 486)	269 (283, 552)
Functional status									
	Working	*n*	346	291	256	214	210	220	211
		Median	157.0	270.0	305.5	324.0	350.5	362.0	371.0
		IQR (Q1, Q3)	105.75 (97.25, 203)	199 (186, 385)	191.25 (215.75, 407)	237.75 (236.25, 474)	258.75 (257, 515.75)	199.75 (282.75, 482.5)	196 (283.5, 479.5)
	Ambulatory	*n*	99	81	64	69	61	59	50
		Median	98.0	232.0	271.5	275.0	290.0	310.0	380.0
		IQR (Q1, Q3)	96 (55, 151)	232 (128, 360)	246.25 (159.75, 406)	315 (161, 476)	302 (169, 471)	253.5 (218.5, 472)	236 (245.5, 481.5)
TB status									
	Negative	*n*	368	311	268	232	231	225	213
		Median	150.0	266.0	306.5	329.5	342.0	357.0	371.0
		IQR (Q1, Q3)	107 (85, 192)	205 (180, 385)	203 (212, 415)	261.25 (220.75, 482)	286.5 (236, 522.5)	209 (270, 479)	190 (281, 471)
	Positive	*n*	77	61	52	51	40	54	48
		Median	132.0	241.0	253.5	297.0	348.5	367.5	404.0
		IQR (Q1, Q3)	98 (84, 182)	202 (162, 364)	207.5 (162, 369.5)	214 (176, 390)	286.25 (179.5, 465.75)	242 (240.5, 482.5)	273 (250, 523)

### 3.2. Exploratory Data Analysis

**Figure 1:**
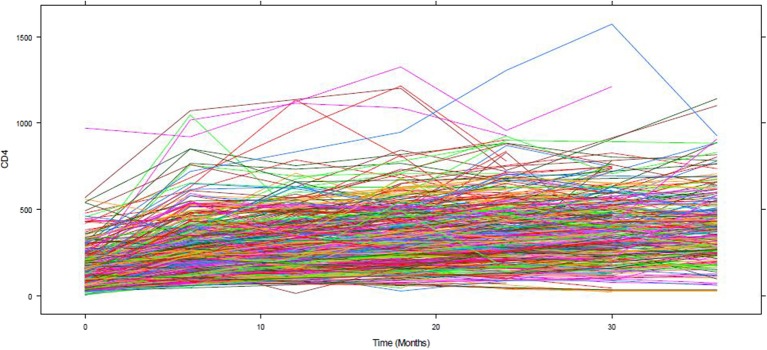
The individual profile plot of CD4 count.

**Figure 2:**
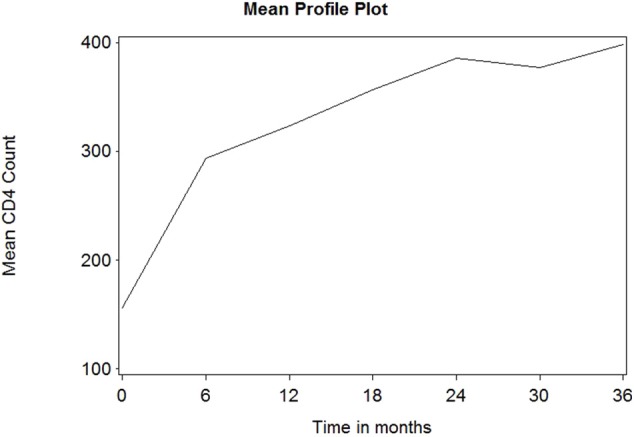
The overall mean profile plot of CD4 cell count.

Figure 1 depicts the individual profile plot of the CD4^+^ cell count of HIV-infected patients included in the study. The plot provides some information on the between patients' CD4^+^ cell count variability and illustrates the over-time change in patients' CD4^+^ cell count. Some individuals have an erratic CD4^+^ cell count and others have a CD4^+^ cell count that slowly increases over time. As one can see from the graph, there is a considerably large difference in the intercepts of individual trajectories. Similarly, some trajectories are steeper, while others were almost horizontal, indicating the possible variability in the slope of CD4^+^ cell counts. Therefore, because of the variability in the intercept and slope of trajectories, using a mixed model could fit the data very well. The overall mean profile plot of the CD4^+^ cell count shows somehow a linear increasing pattern of CD4^+^ cell count over time (Figure 2), suggesting that a linear time effect seems reasonable. The mean CD4^+^ cell count increases at a high rate from baseline till the 6th month and then starts to increase slowly from 6 to 24th month and decreases at month 30.

### 3.3. Model Results

Table 4 summarizes the parameter estimates of Poisson and Poisson-Gamma regression models employed on the CD4^+^ cell count. All parameters included in the Poisson regression model are significant at 5% level of significance. For this study, the data were overdispersed, as the sample variance of CD4^+^ cell count at all time points was greater than its corresponding sample means (Table 2). A likelihood ratio (LR) test was used to test the null hypothesis that the restriction in the Poisson model was true. The test revealed that the null hypothesis was rejected, implying the presence of overdispersion in our data. The Poisson-Gamma model better fits the data as compared to the Poisson model with smallest AIC value. The Poisson-Normal model with both random intercept and slope was found to be the best fit since it has smaller information criteria values compared with the only random intercept model. The parameter estimates of this model are displayed in Table 5. Depending on this model time, the WHO stage and initial CD4^+^ cell count were found to be significant factors of patients' CD4^+^ cell count progression.

**Table 4:** Poisson and Poisson-Gamma models.

	**Poisson**			**Poisson-Gamma**		
**Effect**	**Estimate**	**s.e**	***P*-value**	**Estimate**	**s.e**	***P*-value**
Intercept	4.6438	0.0118	<0.0001	4.5387	0.0954	<0.0001
Time	0.0201	0.0001	<0.0001	0.0248	0.0008	<0.0001
Sex (Ref. = Male)						
Female	0.1418	0.0032	<0.0001	0.1069	0.0260	<0.0001
Marital status (Ref. = Divorced)						
Married	0.0632	0.0031	<0.0001	0.0110	0.0255	0.6647
Never	0.1290	0.0047	<0.0001	0.0853	0.0382	0.0256
Widowed	0.0508	0.0039	<0.0001	0.0065	0.0319	0.8398
Level of education (Ref. = Secondary)						
No	0.0264	0.0033	<0.0001	0.0157	0.0267	0.5577
Primary	−0.0066	0.0034	0.0544	−0.0005	0.0273	0.9857
Tertiary	0.0565	0.0046	<0.0001	0.0245	0.0386	0.5257
Functional status (Ref. = Ambulatory)						
Working	0.0067	0.0033	0.0457	−0.0123	0.0263	0.6397
WHO stage (Ref. = Stage II)						
Stage I	0.0281	0.0046	<0.0001	0.0204	0.0377	0.5882
Stage III	0.1125	0.0035	<0.0001	0.0989	0.0279	0.0004
Stage IV	0.0257	0.0058	<0.0001	0.0061	0.0473	0.8968
TB status (Ref. = Positive)						
Negative	0.0477	0.0034	<0.0001	0.0570	0.0272	0.0364
Age	−0.0038	0.0001	<0.0001	−0.0033	0.0011	0.0040
Weight	0.0036	0.0002	<0.0001	0.0021	0.0013	0.1066
Base CD4	0.0024	0.0000	<0.0001	0.0034	0.0001	<0.0001
Dispersion parameter (1/α)				4.666	0.139	
AIC	145,787			27,848		

**Table 5:** Poisson-Normal model.

	**Random intercept only**			**Random intercept and slope**		
**Effects**	**Estimate**	**s.e**	***P*-value**	**Estimate**	**s.e**	***P*-value**
Intercept	4.5063	0.1637	< 0.0001	4.2967	0.1514	< 0.0001
Time	0.0211	0.0001	< 0.0001	0.0243	0.0008	< 0.0001
Sex (Ref. = Male)						
Female	0.1242	0.0451	0.0060	0.0767	0.0422	0.0692
Marital status (Ref. = Divorced)						
Married	0.0175	0.0443	0.6921	0.0204	0.0408	0.6165
Never	0.1004	0.0656	0.1260	0.0881	0.0607	0.1464
Widowed	0.0340	0.0554	0.5393	0.0543	0.0510	0.2876
Level of education (Ref. = Secondary)						
No	−0.02109	0.0463	0.6487	0.0178	0.0427	0.6768
Primary	−0.0255	0.0474	0.5901	0.0164	0.0437	0.7068
Tertiary	−0.0282	0.0663	0.6702	0.0099	0.0611	0.8710
Functional status (Ref. = Ambulatory)						
Working	−0.0036	0.0449	0.9357	0.0456	0.0415	0.2721
WHO stage (Ref. = Stage II)						
Stage I	0.0361	0.0651	0.5791	−0.0092	0.0600	0.8779
Stage III	0.1194	0.0479	0.0126	0.0964	0.0442	0.0292
Stage IV	−0.2138	0.0631	0.0007	−0.0666	0.0622	0.2844
TB status (Ref. = Positive)						
Negative	0.0730	0.0470	0.1205	0.0671	0.0434	0.1217
Age	−0.0027	0.0020	0.1726	−0.0028	0.0018	0.1224
Weight	0.0022	0.0022	0.3238	0.0031	0.0020	0.1354
Base CD4	0.0033	0.0002	< 0.0001	0.0039	0.0002	< 0.0001
AIC	71,331.7			59,073.9		
BIC	71,434.5			59,188.1		
logLik	−35,647.8			−29,516.9		
Random intercept variance	0.1289			0.1203		
Random slope variance				0.0003		
Cov (random effects)				−0.32		

An improvement in both the Poisson-Gamma and Poisson-Normal models as compared with the Poisson model in fitting the data is an indication of the occurrence of both correlation and overdispersion in the data. The Poisson-Gamma-Normal (Negative Binomial log-linear mixed) model proposed by Booth et al. (9) and Molenberghs et al. (10, 11) was fitted to overcome this problem of correlated and overdispersed count data, and the random intercept Poisson-Gamma-Normal Model is a much better fit because of its lower AIC (27,379.9), BIC (27,488.4), and −2loglikelihood (27,342) values as compared to the Poisson-Normal models (Table 6). Therefore, the final model to fit our data was the random intercept Poisson-Gamma-Normal model. We have also tried the Poison-Gamma-Normal model with different (random) linear slopes for a time, but we found that the Poison-Gamma-Normal with random intercept was better based on information criteria (AIC and BIC).

**Table 6:** Poisson-Gamma-Normal model.

	**Random intercept only**			**Random intercept and slope**		
**Effects**	**Estimate**	**s.e**	***P*-value**	**Estimate**	**s.e**	***P*-value**
Intercept	4.4105	0.1555	< 0.0001	4.3591	0.1078	< 0.0001
Time	0.0243	0.0007	< 0.0001	0.0239	0.0010	< 0.0001
Sex (Ref. = Male)						
Female	0.1147	0.0427	0.0073	0.0743	0.0295	0.0117
Marital status (Ref. = Divorced)						
Married	0.0173	0.0419	0.6790	0.0183	0.0289	0.5277
Never	0.1024	0.0623	0.1001	0.0903	0.0430	0.0358
Widowed	0.0406	0.0524	0.4390	0.0424	0.0362	0.2424
Level of education (Ref. = Secondary)						
No	−0.0012	0.0438	0.9787	0.0256	0.0302	0.3955
Primary	−0.0052	0.0449	0.9070	0.0188	0.0312	0.5475
Tertiary	0.0039	0.0630	0.9507	0.0004	0.0432	0.9928
Functional status (Ref. = Ambulatory)						
Working	0.0139	0.0427	0.7456	0.0282	0.0298	0.3448
WHO stage (Ref. = Stage II)						
Stage I	0.0260	0.0616	0.6726	−0.0055	0.0424	0.8976
Stage III	0.0989	0.0455	0.0299	0.0870	0.0314	0.0056
Stage IV	−0.0136	0.0773	0.8607	−0.0257	0.0544	0.6364
TB status (Ref. = Positive)						
Negative	0.0661	0.0446	0.1385	0.0525	0.0310	0.0898
Age	−0.0027	0.0019	0.1440	−0.0028	0.0013	0.0286
Weight	0.0023	0.0021	0.2721	0.0027	0.0014	0.0612
Base CD4	0.0034	0.0002	< 0.0001	0.0039	0.0001	< 0.0001
Dispersion parameter (1/α)	7.7009			7.7009		
AIC	27,379.9			27,487.7		
BIC	27,488.4			27,607.6		
logLik	−13,671.0			−13,722.8		
Random intercept variance	0.08837			0.0000		
Random slope variance				0.00025		
Cov (random effects)				0		

Based on the results obtained from the Poisson-Gamma-Normal model, time in months, sex, and baseline CD4^+^ cell count were found to be significant factors of the CD4^+^ cell count of a patient (Table 8). For a given patient, keeping the random intercept and other covariates constant, one more month on ART increased the CD4^+^ cell count by a multiplicative factor of *e*^0.0243^ = 1.0246.

**Table 7:** Summary of information criteria of different models.

	**Models**			
**Criteria**	**Poisson**	**Poisson-Normal**	**Poisson-Gamma**	**Poisson-Gamma-Normal**
AIC	145,787	59,073.9	27,848	27,379.9
BIC	145,884	59,188.1	27,951	27,488.4
−2logLik	145,754	59,033.8	27,812	27,342

**Table 8:** Poisson-Gamma-Normal model.

	**Random intercept only**		
**Effects**	**Estimate**	**s.e**	***P*-value**
Intercept	4.4105	0.1555	< 0.0001
Time	0.0243	0.0007	< 0.0001
Sex (Ref. = Male)			
Female	0.1147	0.0427	0.0073
Marital status (Ref. = Divorced)			
Married	0.0173	0.0419	0.6790
Never	0.1024	0.0623	0.1001
Widowed	0.0406	0.0524	0.4390
Level of education (Ref. = Secondary)			
No	−0.0012	0.0438	0.9787
Primary	−0.0052	0.0449	0.9070
Tertiary	0.0039	0.0630	0.9507
Functional status (Ref. = Ambulatory)			
Working	0.0139	0.0427	0.7456
WHO stage (Ref. = Stage II)			
Stage I	0.0260	0.0616	0.6726
Stage III	0.0989	0.0455	0.0297
Stage IV	−0.0136	0.0773	0.8607
TB status (Ref. = Positive)			
Negative	0.0661	0.0446	0.1385
Age	−0.0027	0.0019	0.1440
Weight	0.0023	0.0021	0.2721
Base CD4	0.0034	0.0002	< 0.0001
Dispersion parameter (1/α)	7.7009		
Random intercept variance	0.08837		

A female patient had a CD4^+^ cell count of 1.1215 times that of a male patient, adjusting for other covariates and random intercept. A unit change in baseline CD4^+^ cell count increased the CD4^+^ cell count of a patient by a factor of 1.0034, fixing the values of the other covariates and the random intercept constant.

The dispersion parameter (1/α) has been estimated, in the final model, as 7.7009, and the Gamma (overdispersion) random effects are assumed to follow a Gamma distribution with unit mean and shape parameter α (0.130).

### 3.4. Discussion

The effects of demographic and clinical factors on the progression of CD4^+^ cell counts over time of HIV patients taking ART treatment in Debre Markos Referral Hospital were assessed using Poisson longitudinal models since the response variable of interest CD4^+^ cell count is a count variable.

The results of the summary statistics revealed that the value of IQR is high at all time points, which might be an indication for high variation among the patients' CD4^+^ cell count at baseline as well as at different time points after the initiation of ART treatment. This variation might have been caused by the year at which the patients started ART treatment, as there have been different WHO's CD4^+^ cell count cut-off points to initiate ART treatment at different times. Although most of the patients included in our study started with lower CD4^+^ cell counts (<200 cells/mm^3^), there were patients who had higher baseline CD4^+^ cell counts (971 cells/mm^3^). Despite the continuous effort to initiate early, some patients still presented with lower CD4^+^ cell counts, which might be due to patients' lack of willingness to get tested (13, 14) or difficulties to provide treatments to all patients in lower-income countries including Ethiopia. Hence, we believe that our result could be generalizable. The final model also indicated that initial CD4^+^ cell count (CD4^+^ cell count at the start of the treatment) significantly affects CD4 count progression. Therefore, based on our findings we recommend patients to start the treatment early as of the WHO's “treat all” recommendation.

The sign of the parameter estimate of WHO stage III is positive, which implies that a patient with WHO stage III has a higher CD4^+^ cell count as compared with a patient of WHO stage II. It might be because the number of patients with WHO stage III are much higher (non-comparable) than patients with WHO stage II. The relationship between CD4^+^ cell count and WHO stage III might also be explained by the baseline CD4^+^ cell count. Duration of treatment also have a positive effect on the CD4^+^ cell count progression of HIV patients. This means patients with longer time on ART treatment have good recovery of CD4^+^ cell count than that of patients with short duration on the treatment.

## 4. Conclusion

An analysis of CD4^+^ cell count data using conventional models like linear mixed models might be inadequate as the data were highly skewed and may not satisfy normality (multivariate) assumption as demonstrated in our data.

In this study, CD4^+^ cell count data of 445 HIV patients under ART in Debre Markos Referral Hospital was analyzed using different longitudinal count models, and the Poisson-Gamma-Normal model was selected as the final model to fit the data based on different selection criteria. The Poisson-Gamma-Normal model handles overdispersion and correlation simultaneously.

The duration on ART treatment (time in months), sex of patients, and baseline CD4^+^ cell count were all identified as potential risk factors of CD4^+^ cell count progression. Having a good CD4^+^ cell count at baseline had a positive impact on CD4^+^ cell count evolution over time.

Although good CD4^+^ cell count progress in response to ART was observed, most of the patients (78.0%) were at decreased CD4^+^ cell counts (<200 cells/mm^3^) when enrolled for ART treatment, which might have contributed to low CD4^+^ count recovery in some patients.

## 5. Limitations and Recommendation

In our study, we only considered patients from one hospital. The likelihood inference of the models considered in this study are valid under MCAR (missing completely at random). In the current study, we did not carry out a sensitivity analysis, and we only considered linear slopes models, although a different linear slope for different time periods seems reasonable. Hence, we recommend that researchers consider sensitivity analysis and data obtained from different Hospitals. The age and weight of patients might have a non-linear relationship with the CD4^+^ cell count. We recommend smoothing techniques like splines to be explored for further studies. The assumption of multivariate normality that is assumed by most statistical models used in longitudinal data analysis should be checked before analysis. Efficient methods like the ones used in this study could be considered if the assumption is violated.

## Data Availability Statement

The datasets generated for this study are available on request to the corresponding author.

## Ethics Statement

Before data collection, a letter of support written by the Statistics Department of Addis Ababa University was submitted to Debre Markos Hospital and permission to collect anonymized data was obtained. The data was extracted by trained data clerks in the ART Clinic and none of the researchers had access to original cards of patients. Written informed consent for participation was not required for this study in accordance with the national legislation and the institutional requirements.

## Author Contributions

BAn conceived the idea, performed the data cleaning and analysis, interpreted the ensuing results, and drafted the manuscript. BAy supervised the study, contributed to the conception, and revised the manuscript. Both the authors read and approved the final draft.

### Conflict of Interest

The authors declare that the research was conducted in the absence of any commercial or financial relationships that could be construed as a potential conflict of interest.

## Supplementary Material

R codes used to fit the models are attached as supplemental data.
