# Supplementary material for: Progression of HIV Disease Among Patients on ART in Ethiopia: Application of Longitudinal Count Models
Source: Front Public Health. 2020 Feb 19;7:415. doi: 10.3389/fpubh.2019.00415 (PMC7042380; doi:10.3389/fpubh.2019.00415)
Supplement: Supplementary file 1 [file Presentation_1.PPTX]

## Slide 1
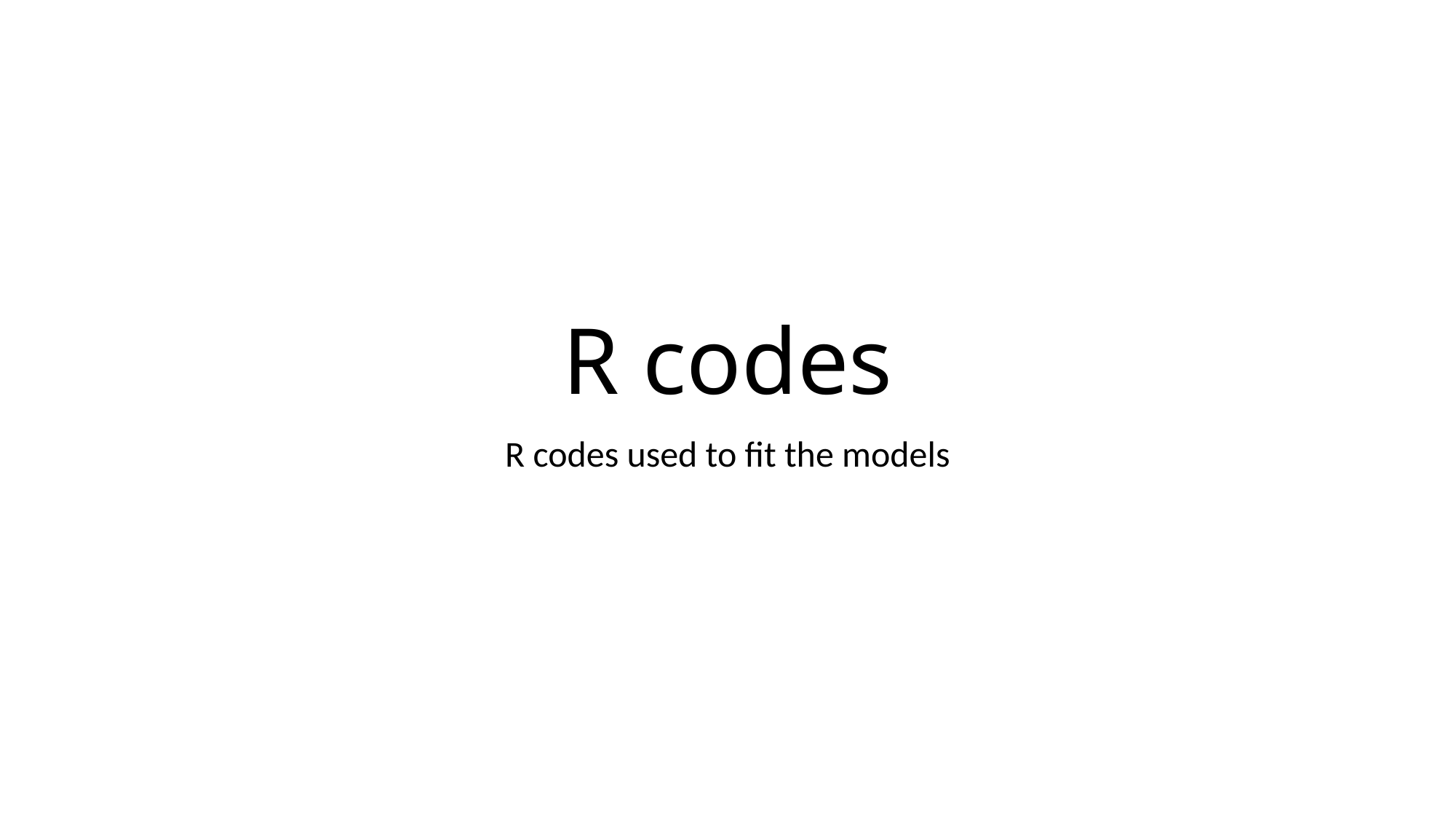

# R codes
R codes used to fit the models

## Slide 2
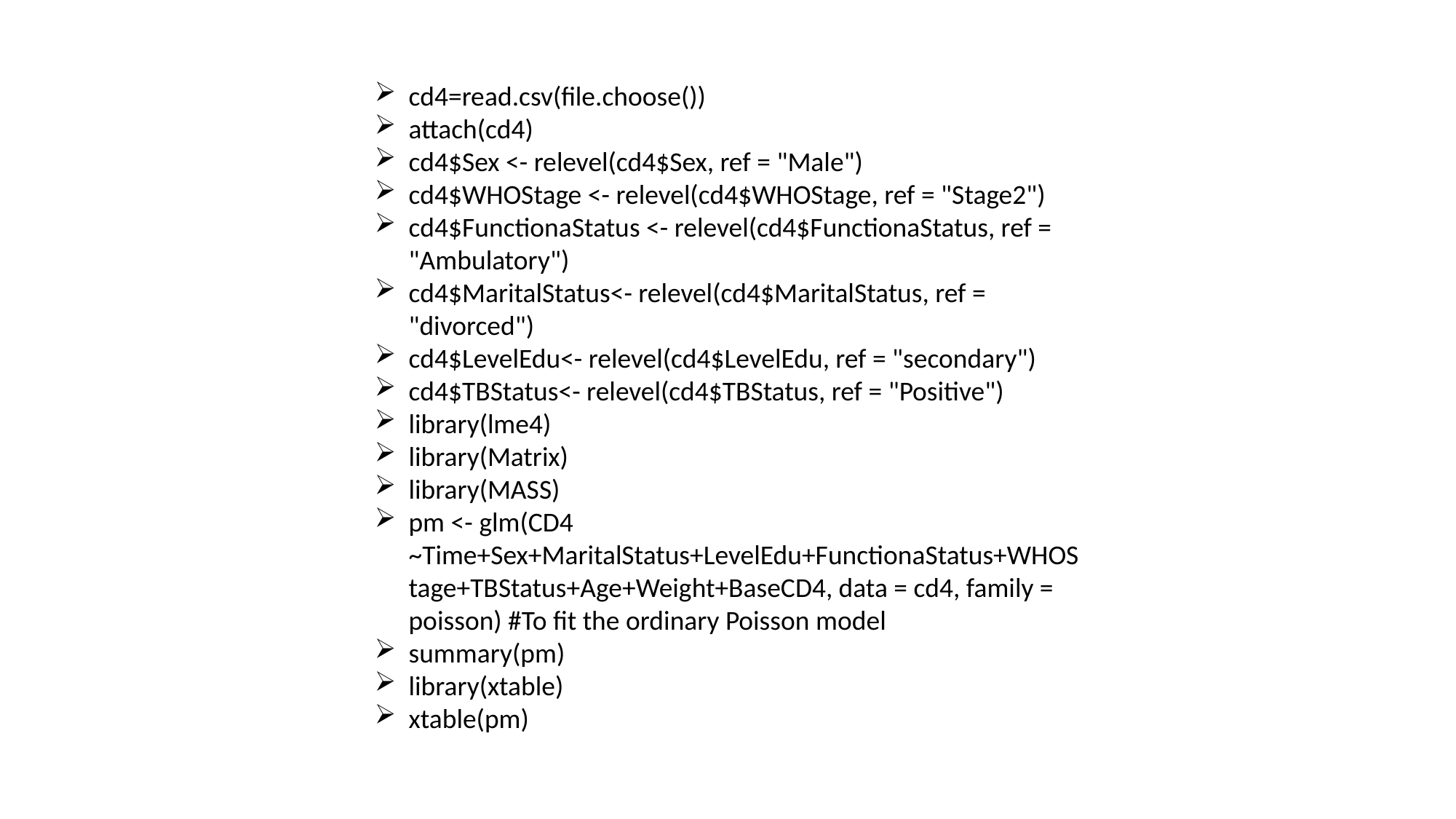

cd4=read.csv(file.choose())
attach(cd4)
cd4$Sex <- relevel(cd4$Sex, ref = "Male")
cd4$WHOStage <- relevel(cd4$WHOStage, ref = "Stage2")
cd4$FunctionaStatus <- relevel(cd4$FunctionaStatus, ref = "Ambulatory")
cd4$MaritalStatus<- relevel(cd4$MaritalStatus, ref = "divorced")
cd4$LevelEdu<- relevel(cd4$LevelEdu, ref = "secondary")
cd4$TBStatus<- relevel(cd4$TBStatus, ref = "Positive")
library(lme4)
library(Matrix)
library(MASS)
pm <- glm(CD4 ~Time+Sex+MaritalStatus+LevelEdu+FunctionaStatus+WHOStage+TBStatus+Age+Weight+BaseCD4, data = cd4, family = poisson) #To fit the ordinary Poisson model
summary(pm)
library(xtable)
xtable(pm)

## Slide 3
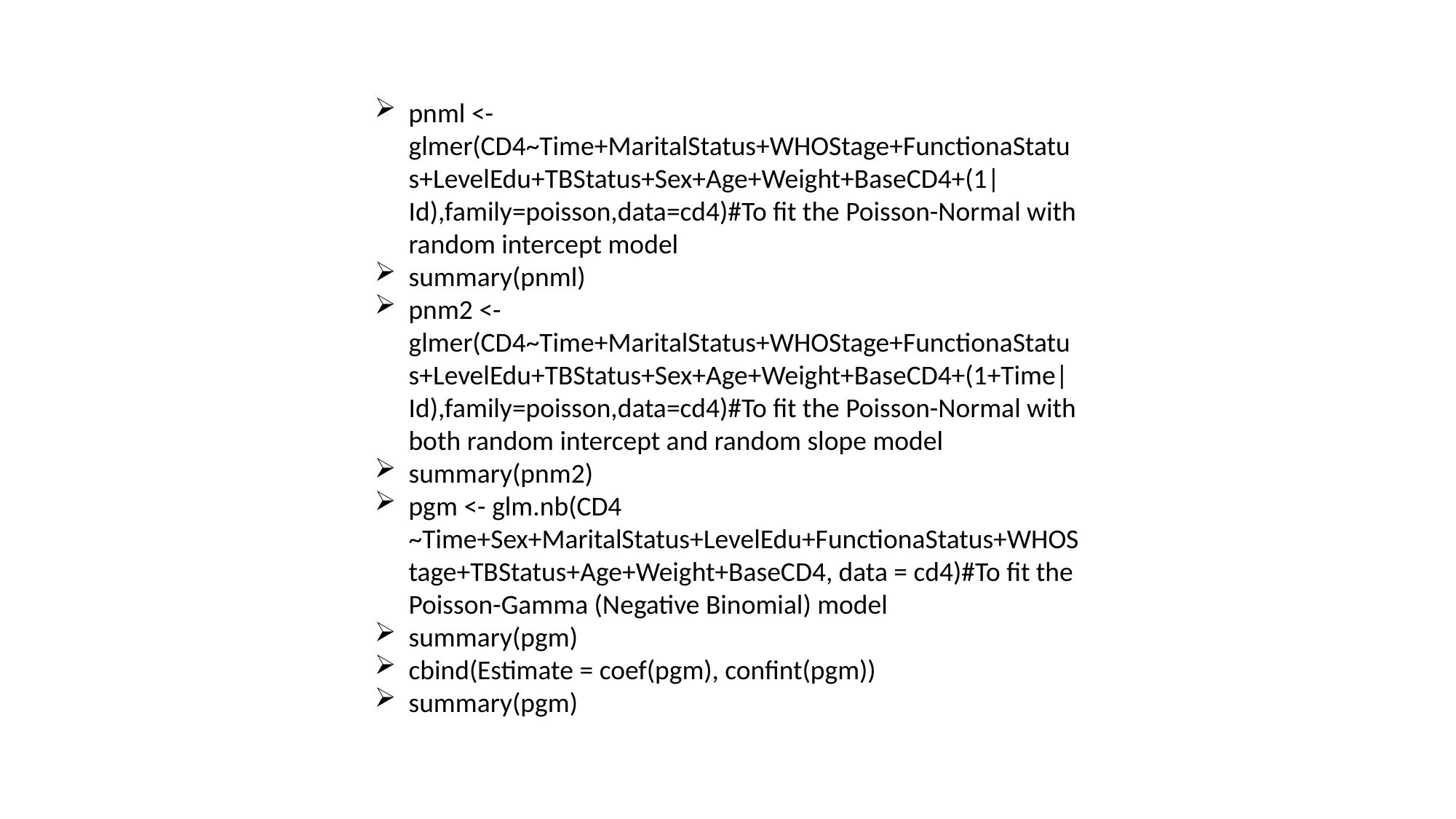

pnml <- glmer(CD4~Time+MaritalStatus+WHOStage+FunctionaStatus+LevelEdu+TBStatus+Sex+Age+Weight+BaseCD4+(1|Id),family=poisson,data=cd4)#To fit the Poisson-Normal with random intercept model
summary(pnml)
pnm2 <- glmer(CD4~Time+MaritalStatus+WHOStage+FunctionaStatus+LevelEdu+TBStatus+Sex+Age+Weight+BaseCD4+(1+Time|Id),family=poisson,data=cd4)#To fit the Poisson-Normal with both random intercept and random slope model
summary(pnm2)
pgm <- glm.nb(CD4 ~Time+Sex+MaritalStatus+LevelEdu+FunctionaStatus+WHOStage+TBStatus+Age+Weight+BaseCD4, data = cd4)#To fit the Poisson-Gamma (Negative Binomial) model
summary(pgm)
cbind(Estimate = coef(pgm), confint(pgm))
summary(pgm)

## Slide 4
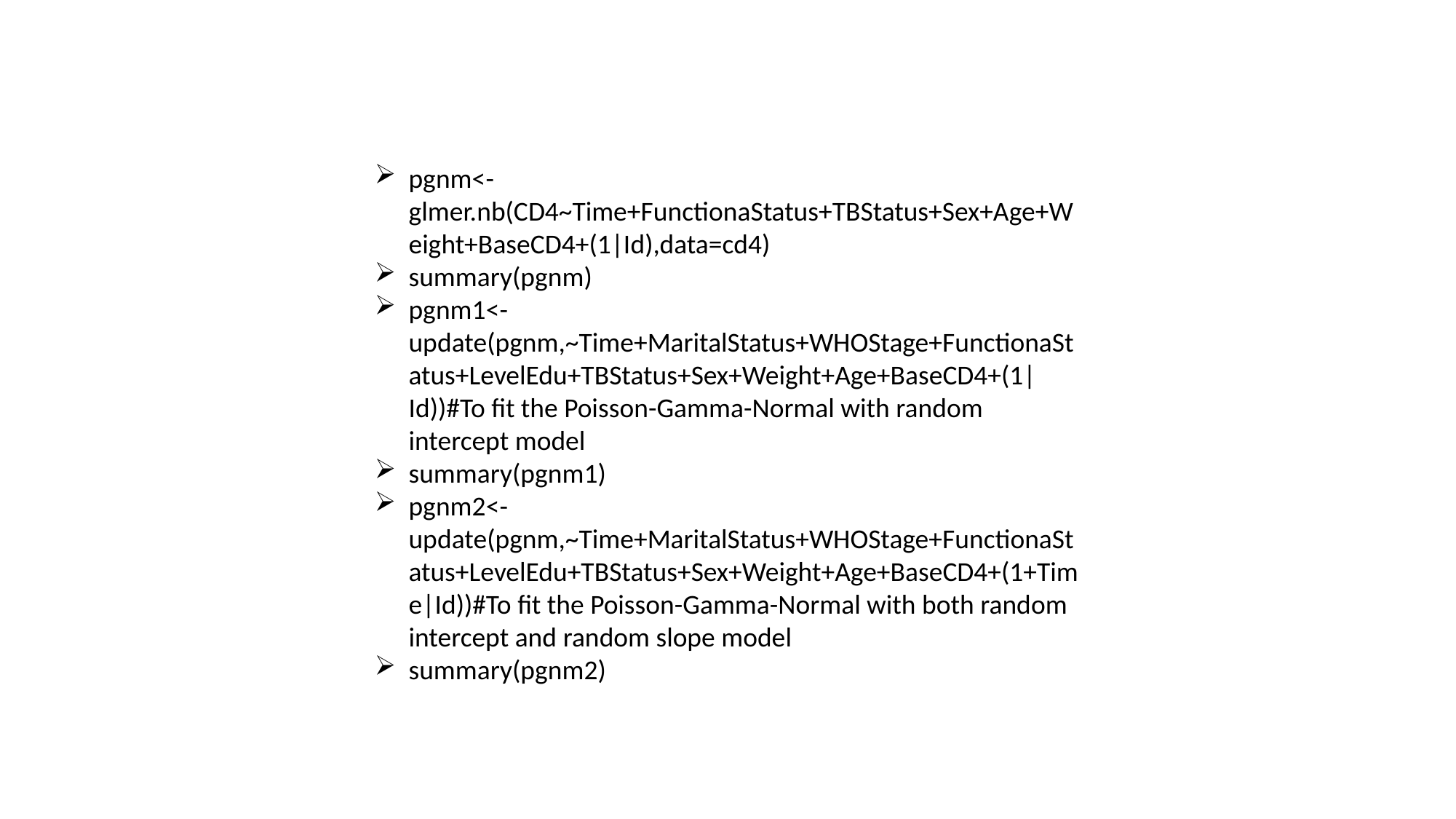

pgnm<- glmer.nb(CD4~Time+FunctionaStatus+TBStatus+Sex+Age+Weight+BaseCD4+(1|Id),data=cd4)
summary(pgnm)
pgnm1<-update(pgnm,~Time+MaritalStatus+WHOStage+FunctionaStatus+LevelEdu+TBStatus+Sex+Weight+Age+BaseCD4+(1|Id))#To fit the Poisson-Gamma-Normal with random intercept model
summary(pgnm1)
pgnm2<-update(pgnm,~Time+MaritalStatus+WHOStage+FunctionaStatus+LevelEdu+TBStatus+Sex+Weight+Age+BaseCD4+(1+Time|Id))#To fit the Poisson-Gamma-Normal with both random intercept and random slope model
summary(pgnm2)
